# Pim1 promotes human prostate cancer cell tumorigenicity and c-MYC transcriptional activity

**DOI:** 10.1186/1471-2407-10-248

**Published:** 2010-06-01

**Authors:** Jongchan Kim, Meejeon Roh, Sarki A Abdulkadir

**Affiliations:** 1 Department of Pathology, Vanderbilt University Medical Center, Nashville, TN, USA

## Abstract

**Background:**

The serine/threonine kinase PIM1 has been implicated as an oncogene in various human cancers including lymphomas, gastric, colorectal and prostate carcinomas. In mouse models, Pim1 is known to cooperate with c-Myc to promote tumorigenicity. However, there has been limited analysis of the tumorigenic potential of Pim1 overexpression in benign and malignant human prostate cancer cells *in vivo*.

**Methods:**

We overexpressed Pim1 in three human prostate cell lines representing different disease stages including benign (RWPE1), androgen-dependent cancer (LNCaP) and androgen-independent cancer (DU145). We then analyzed *in vitro *and *in vivo *tumorigenicity as well as the effect of Pim1 overexpression on c-MYC transcriptional activity by reporter assays and gene expression profiling using an inducible MYC-ER system. To validate that Pim1 induces tumorigenicity and target gene expression by modulating c-MYC transcriptional activity, we inhibited c-MYC using a small molecule inhibitor (10058-F4) or RNA interference.

**Results:**

Overexpression of Pim1 alone was not sufficient to convert the benign RWPE1 cell to malignancy although it enhanced their proliferation rates when grown as xenografts *in vivo*. However, Pim1 expression enhanced the *in vitro *and *in vivo *tumorigenic potentials of the human prostate cancer cell lines LNCaP and DU145. Reporter assays revealed increased c-MYC transcriptional activity in Pim1-expressing cells and mRNA expression profiling demonstrated that a large fraction of c-MYC target genes were also regulated by Pim1 expression. The c-MYC inhibitor 10058-F4 suppressed the tumorigenicity of Pim1-expressing prostate cancer cells. Interestingly, 10058-F4 treatment also led to a reduction of Pim1 protein but not mRNA. Knocking-down c-MYC using short hairpin RNA reversed the effects of Pim1 on Pim1/MYC target genes.

**Conclusion:**

Our results suggest an *in vivo *role of Pim1 in promoting prostate tumorigenesis although it displayed distinct oncogenic activities depending on the disease stage of the cell line. Pim1 promotes tumorigenicity at least in part by enhancing c-MYC transcriptional activity. We also made the novel discovery that treatment of cells with the c-MYC inhibitor 10058-F4 leads to a reduction in Pim1 protein levels.

## Background

Pim1 is a constitutively active serine/threonine kinase [1], whose activity is therefore primarily regulated at the level of expression and stability. Pim1 enhances cell cycle progression by phosphorylating Cdc25A, Cdc25C, p21^cip1^, p27^kip1 ^and c-Tak1 [2-5] or by associating with protein complexes required for mitosis [6]. Pim1 also inhibits apoptosis by phosphorylating apoptotic proteins including Bad [7], FOXO3a [5] and ASK1 [8]. PIM1 has been implicated as an oncogene whose expression is dysregulated in several human cancers including lymphomas, gastric, colorectal and prostate cancers [9].

The oncogenic activity of Pim1 was first discovered in lymphomagenesis. *PIM1 *was identified as a non-immunoglobulin (IG)/BCL6 translocation partner gene and 6p21, its chromosomal locus, was amplified in B-cell lymphomas [10,11]. *PIM1 *is also known to be a target locus for aberrant somatic hypermutation in some lymphomas [12-15]. Eμ-*Pim1 *transgenic mice engineered to overexpress Pim1 in lymphocytes develop T cell lymphomas and cooperate with another proto-oncogene *Myc *to accelerate the disease progression [16-18].

In human prostate cancer, PIM1 expression is known to be elevated in ~50% of human prostate cancer specimens and its cooperation with MYC was also proposed [19]. Prostate cancer induced by mouse prostate-specific overexpression of *c-MYC *oncogene demonstrated Pim1 mRNA upregulation, suggesting possible synergistic effect between two oncogenes [20]. However, the oncogenic activity of Pim1 itself in prostate cancer using *in vivo *models has not been fully characterized. One study used PC3 human prostate carcinoma cells to show that Pim1 overexpression accelerates tumorigenicity in these cells associated with elevated levels of c-MYC and the phosphorylation of proteins involved in protein synthesis [21]. Here we sought to determine the effects of Pim1 overexpression on the tumorigenic potential of human prostate cells representing distinct stages of disease progression, including benign/non-tumorigenic, tumorigenic/androgen-sensitive and tumorigenic/androgen-independent stages. Using these cells, we analyzed the effects of Pim1 on *in vitro *and *in vivo *tumorigenicity as well as c-MYC transcriptional activity.

## Methods

### Cell lines and cell culture

Cell lines were obtained from American Type Culture Collection. Vector control, Pim1 or kinase dead mutant Pim1 (K67M)-overexpressing cells were generated as described [22]. pBabe-Puro-MYC-ER plasmid (gift from Dr. Gerard Evan, University of California at San Francisco, CA, USA) was used to generate retroviruses and infect RWPE1-Neo and RWPE1-Pim1 cells to generate RWPE1-Neo/MYC-ER and RWPE1-Pim1/MYC-ER cells and the cells were maintained as described [23]. To activate c-MYC in chimeric MYC-ER protein, 100 nM of 4-hydroxytamoxifen (4OHT) in ethanol was added to the cells. LNCaP and DU145 cells were maintained in RPMI with 10% fetal bovine serum.

### Western blot analyses

Western blotting was performed as described [22] using following antibodies: anti-Pim1 (mouse, 1:500, Santa Cruz), anti-beta-Actin (goat, 1:1000, Santa Cruz), anti-phospho-p21 (rabbit, 1:1000, Santa Cruz), anti-p21 (mouse, 1:1000, Santa Cruz), anti-cyclin E (rabbit, 1:1000, Santa Cruz), anti-androgen receptor (rabbit, 1:500, Santa Cruz) and anti-c-Myc (mouse, 1:500, Santa Cruz).

### Cell cycle analysis

Cell cycle was analyzed by fluorescence-activated cell sorting (FACS) as described [24].

### MTT cell proliferation assay

MTT Cell Proliferation Assay Kit was purchased from ATCC and assay was performed in accordance with the manufacturer's protocol. Individual absorbance was measured with plate photometer (Bio-tek).

### Soft agar assays

These were performed as described [25]. 100 × 10^3 ^and 20 × 10^3 ^of LNCaP and DU145 cells were used, respectively and total number of colonies (≥ cutoff sizes) was counted. For c-Myc inhibitor (10058-F4) treatment, 50 × 10^3 ^of LNCaP and DU145 were used and 100 uM 10058-F4 in 0.25% DMSO or 0.25% DMSO alone was added. Three random low power view-fields were selected and each number of colonies (≥ cutoff sizes) was added.

### Xenografts in nude mice

Male nude (nu/nu) mice were obtained from Charles River laboratories. 3 × 10^6^, 3 × 10^6 ^and 5 × 10^6 ^of RWPE1, LNCaP and DU145 cells were mixed with 200 μl of Matrigel (BD Biosciences), respectively. Cells were injected subcutaneously in both flanks of nude mice. Tumor volumes and cross-sectional areas were calculated as described [26,27]. Animal care and experiments were carried out according to the protocols approved by the Institutional Animal Care and Use Committees at Vanderbilt University.

### Histology and immunohistochemical analyses

Nude mice were sacrificed after 30-38 (RWPE1) or 6-8 (LNCaP and DU145) weeks. Xenografted lesions were taken, photographed, fixed in formalin (10%) and embedded in paraffin for subsequent histology as described [28]. Immunohistochemical analyses were performed as described [28] using following antibodies: activated Caspase 3 (rabbit, 1:500, Cell Signaling), Ki67 (rabbit, 1:50, abcam) and phospho-Histone H3 (rabbit, 1:500, Upstate).

### Analysis of androgen-dependent proliferation

15,000 LNCaP cells were plated on 24 well plates. The next day, cells were washed with phosphate-buffered saline (PBS) and phenol red-free RPMI media with charcoal-striped serum was added for androgen starvation. After 2-day starvation, DHT (5α-Dihydrotestosterone) or carrier (ethanol) was added. MTT assays were performed from next day (Day 1). MTT values of DHT-treated cells were divided by those of carrier-untreated cells and triplicate data per group were analyzed.

### DHT treatment and quantitative RT-PCR

For DHT treatment, LNCaP cells were washed with phosphate-buffered saline (PBS) and phenol red-free RPMI media with charcoal-striped serum was added for androgen starvation. After 2-day starvation, DHT (0.1, 1, 10 and 100 nM) or carrier (ethanol) was added and the cells were incubated for 2 days. Total RNA isolation, reverse transcription and subsequent quantitative PCR were performed as described [24]. The following primers were used: Prostate specific antigen (PSA) forward (5'-CAA CCC TGG ACC TCA CAC CTA-3'), PSA reverse (5'-GGA AAT GAC CAG GCC AAG AC-3'); human GAPDH forward (5'-ATG GAA ATC CCA TCA CCA TCT T-3'), human GAPDH reverse (5'-CGC CCC ACT TGA TTT TGG-3'); LAMC2 forward (5'-GGA TGA GAA TCC TGA CAT TGA GTG T-3'), LAMC2 reverse (5'-GTC GTG CGG ATC GTT GTA GA-3'); MT1F forward (5'-ACC TGC CCC ACT GCT TCT T-3'), MT1F reverse (5'-TTG CAA GCC GAG GAG AGA CT-3'); UPP1 forward (5'-TCT GGA GGC AGC CTA TGC A-3'), UPP1 reverse (5'-GCA AAC ACC GAG GAC TCC AT-3'); CDKN1C forward (5'-GCC TCT GAT CTC CGA TTT CTT C-3'), CDKN1C reverse (5'-CAT CGC CCG ACG ACT TCT-3'); CUL3 forward (5'-AGA TTT TGA GGC TCC TTT TTT GG-3'), CUL3 reverse (5'-AAA ATT TCT GGC TTT CCA TCT GAA-3'); SOD2 forward (5'-GCT GCA CCA CAG CAA GCA-3'), SOD2 reverse (5'-TCG GTG ACG TTC AGG TTG TTC-3'); VAV3 forward (5'-CTG GTG AAC AAG GGA CAC TCA A-3'), VAV3 reverse (5'-TTT AGG AGT TCT TCG CAG TCC ATT-3'); mouse Pim1 (5'-ATT CCG TTT GAG CAC GAT GAA-3'), mouse Pim1 reverse (5'-TGA AGA GAC AGT TTG CCT GAA GAA-3'). Each mRNA expression was normalized with GAPDH expression.

### Luciferase assay

300,000 RWPE1 cells were plated on 12 well plates and grown without supplements (bovine pituitary extract and epidermal growth factor) for 24 hours. Transient transfection was performed with the following plasmids: c-Myc-responsive 4× E-box reporter (gift from Dr. Stephen Hann, Vanderbilt University, TN, USA), pMSCV-MYC and pMSCV empty vector. After 30 hours, cell lysates were harvested and luciferase activity was measured using luciferase assay system (Promega). Luciferase activity was normalized to protein concentration and triplicate data per group were analyzed.

### c-Myc inhibitor treatment

c-Myc inhibitor (10058-F4) was purchased from Sigma. Cells were plated on 6-well plates and 50, 100 and 200 uM 10058-F4 in 0.5% DMSO or 0.5% DMSO alone was added on next day when cells were sub-confluent. Cells were harvested after 20 hours and RT-PCR and western blotting were performed from the isolated mRNA and cell lysates, respectively.

### c-Myc gene silencing with small hairpin RNA (shRNAmir)

Control and Pim1-expressing LNCaP cells were plated on 60 mm dishes. Lentiviral shRNAmirs (Open Biosystems) against c-MYC and pGIPZ control plasmid were transiently transfected. Transfection efficiency was monitored by GFP fluorescence. After 3 days, cells were harvested, and mRNA isolation followed by reverse transcription and qRT-PCR and western blotting were performed.

### Genechip analysis

Total RNA was isolated from RWPE1-Neo/MYC-ER and RWPE1-Pim1/MYC-ER cells with and without 24 hr 4OHT treatment. Genechip analysis was performed in duplicates according to manufacturer's protocol (Affymetrix) using U133A chips. Genes whose expressions were altered at least 1.4-fold with significance (*P *< 0.005) in pairwise comparisons were identified.

### Statistical analyses

Each group was compared using t-test. Values are considered statistically significant at *P *< 0.05. Quantitative variables are expressed as means + standard deviation while categorical variables are expressed as numbers (%).

## Results

### Generation of human prostate cell lines at different disease stages, with stable Pim1 overexpression

**Figure 1:**
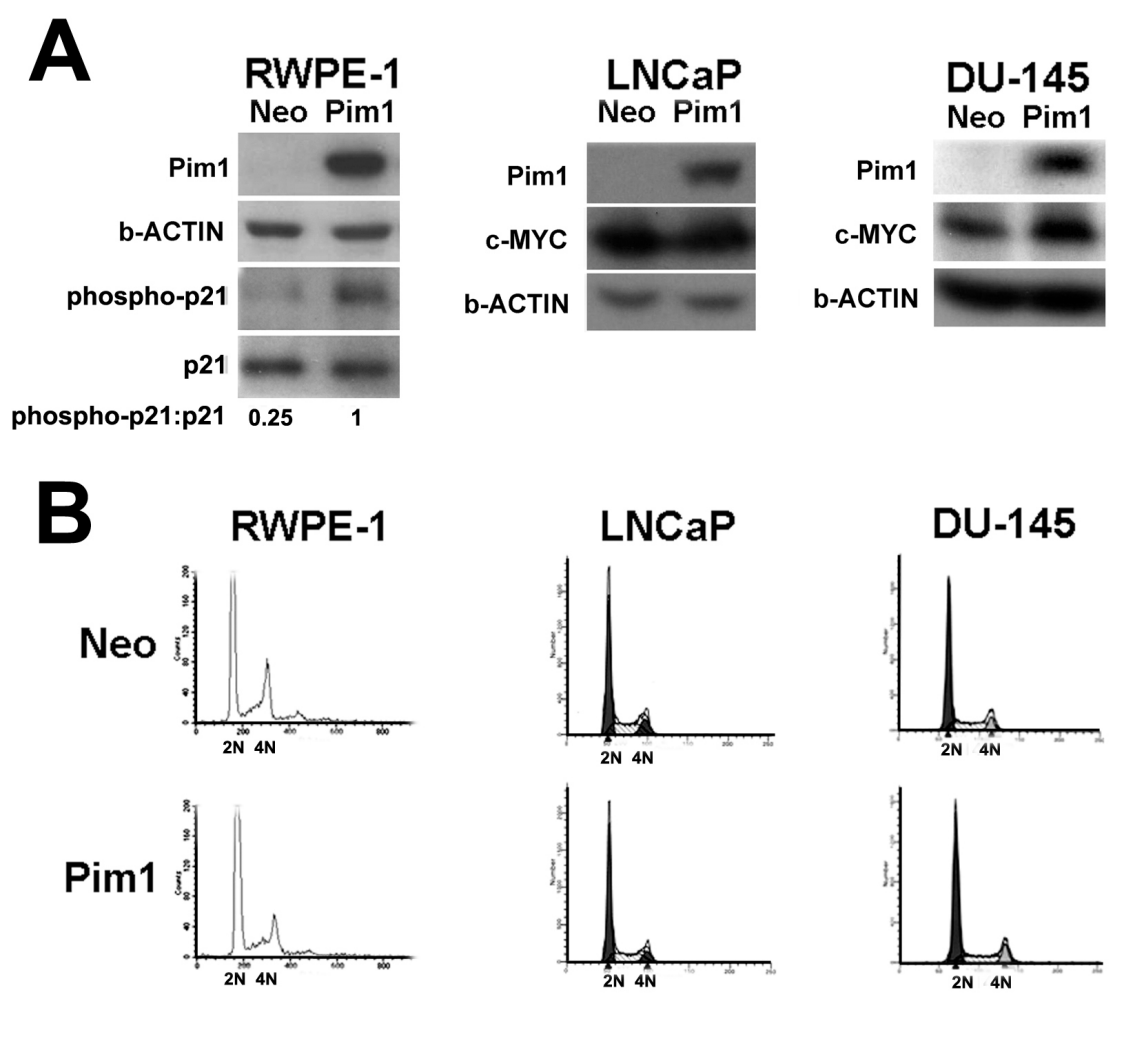
**Overexpression of Pim1 in human prostate cell lines**. (A) Western blots demonstrated Pim1 expression in benign human prostate cell line (RWPE1) and human prostate cancer cell lines (LNCaP and DU145). In addition, endogenous levels of c-MYC were upregulated in two cancer cell lines. Phosphorylation of p21 was increased in Pim1-expressing RWPE1 cells compared to control cells (Neo). (B) Cell sorting analyses showed that there was no difference between control (Neo) and Pim1 cells in cell cycle at the time when the cells were grafted.

**Figure 2:**
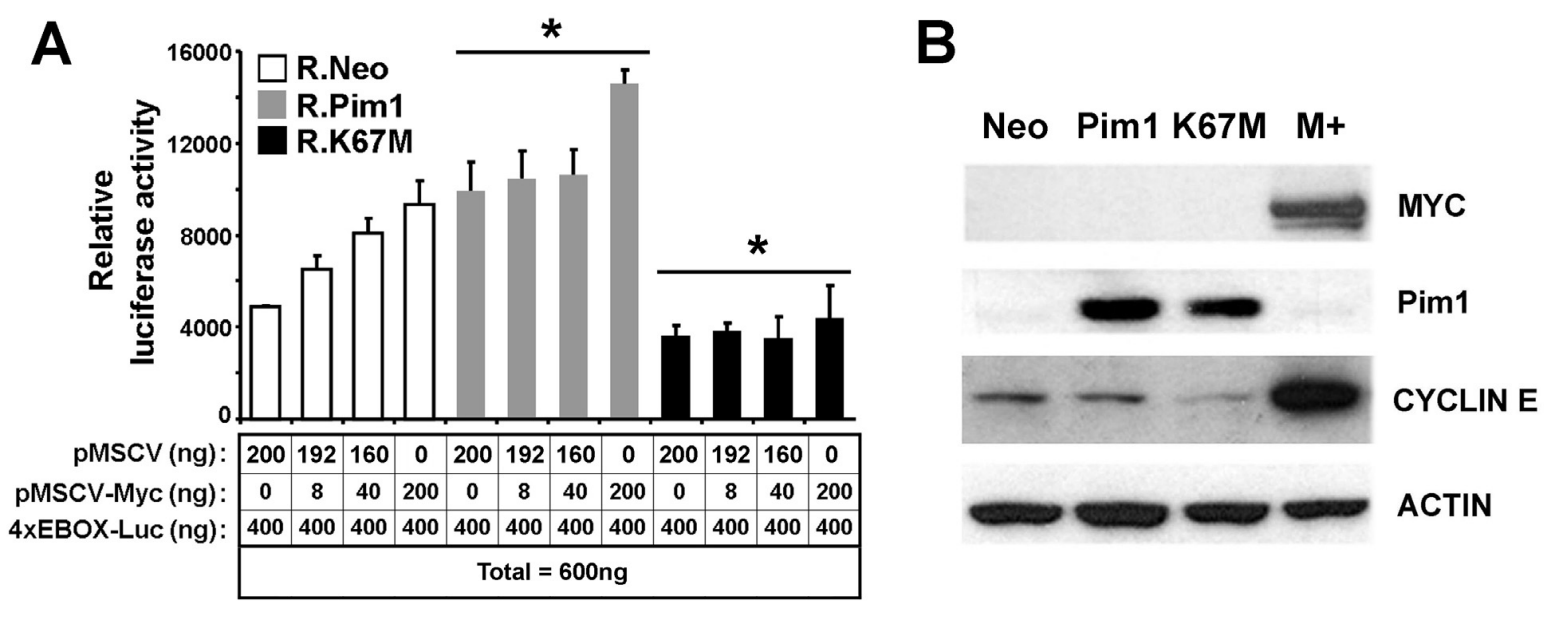
**Increase in c-MYC activity due to Pim1 overexpression**. (A) Luciferase assay using c-MYC responsive reporter construct demonstrated that Pim1 overexpression induced transcriptional activity of c-MYC but its kinase dead mutant Pim1 (K67 M) showed dramatically repressed c-MYC activity compared to control. *P < 0.05. (B) Western blots showed c-MYC expression is undetectable in RWPE1 cells.

To examine the effects of Pim1 overexpression on prostate tumorigenesis, we selected three human prostate cell lines at different cancer disease stages: RWPE1, an immortalized, benign, androgen-responsive human prostatic epithelial cell line [29]; LNCaP, a tumorigenic, androgen-responsive human prostate cancer cell line [30,31]; and DU145, a tumorigenic androgen-independent human prostate cancer cell line [32-34]. We stably expressed Pim1 in all the cell lines and established pools of Pim-1 expressing and vector control (Neo) cells. LNCaP and DU145 cells express high levels of c-MYC endogenously (Figure 1A) but c-MYC levels in the RWPE1 cells were not detectable by western blot (see Figure 2B). To examine Pim1 activity, we assessed a known phosphorylation substrate of Pim1, p21 using a phospho-specific antibody [3]. Phospho-p21 levels were increased 4-fold in RWPE1-Pim1 cells (Figure 1A), showing increased kinase activity of Pim1.

Pim1 has been reported to induce polyploidy and chromosomal instability (CIN) in a passage-dependent manner [22,24] and the resultant polyploidy/CIN was shown to increase *in vitro and in vivo *tumorigenicity [25]. We therefore examined the cell cycle profiles of the cells we used in the tumorigenicity assays in the current study to ensure that we utilize cells that have not progressed to polyploidy. As shown in Figure 1B, there were no differences in ploidy between control and Pim1-expressing cells and this is probably due to relatively early passage number (RWPE1, passage 18; LNCaP, passage 27; DU145, passage 23).

### Pim1 promotes proliferation and attenuates apoptosis of RWPE1 cells *in vivo *without malignant conversion

**Figure 3:**
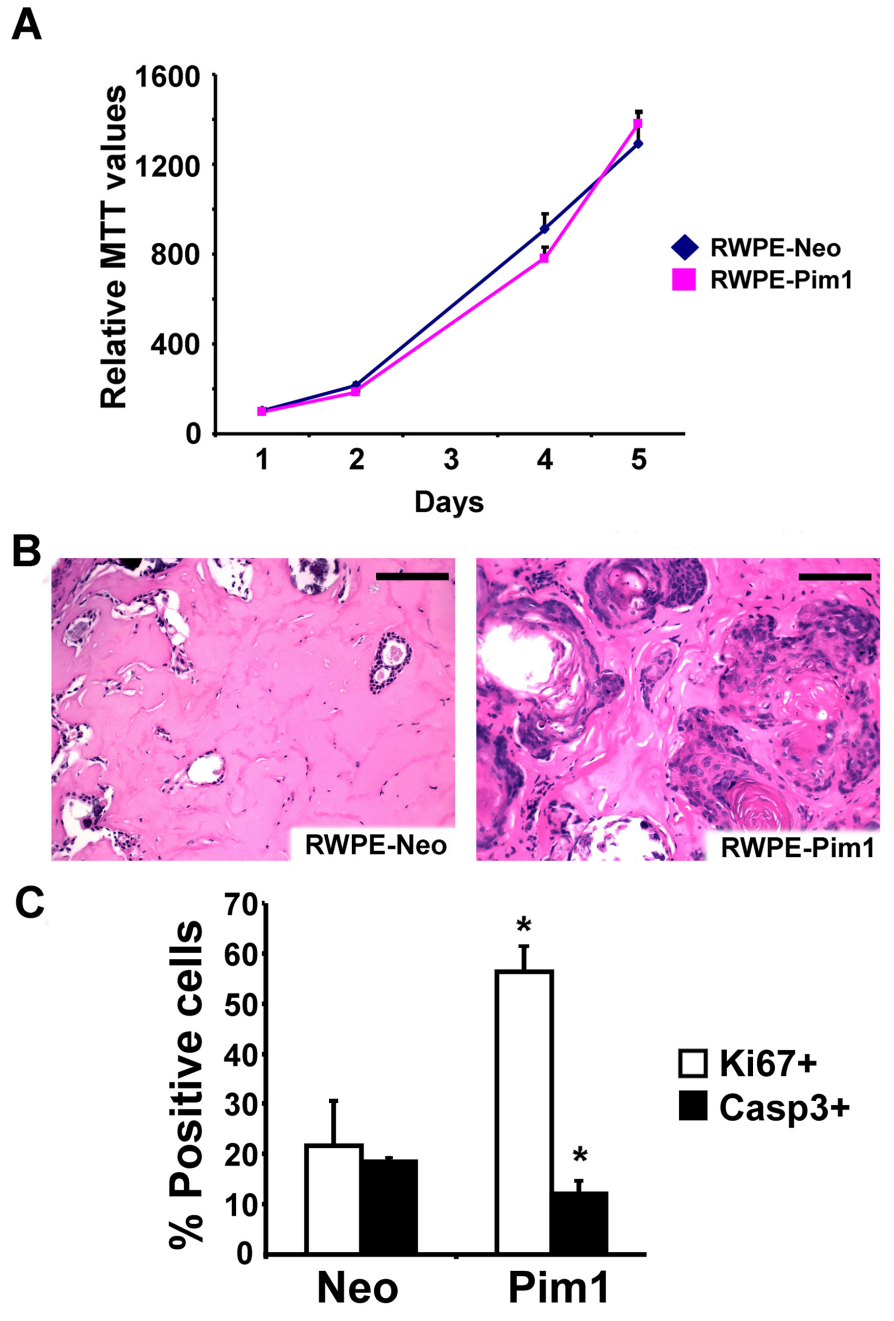
**Pim1 expression is insufficient to convert benign human prostate cells (RWPE1) to malignancy**. (A) 3-(4,5-dimethylthiazol-2-yl)-2,5-diphenyltetrazolium bromide (MTT) assay showed there was no difference in *in vitro *cell proliferation between control and Pim1-expressing RWPE1 cells. (B) Representative H&E images of grafts. RWPE1-Neo (N = 5); RWPE1-Pim1 (N = 7). Scale bars: 100 μm. (C) Quantitation of proliferation and apoptosis in xenografts after immunostaining for Ki67 and activated Caspase 3, respectively. *P < 0.05.

*In vitro *cell proliferation (MTT) assay showed that there was no discernible change in cellular proliferation due to Pim1 expression in RWPE1 cells (Figure 3A). In addition, RWPE1-Pim1 cells failed to form colonies in soft agar assay (data not shown), verifying that *in vitro *tumorigenicity of Pim1-expressing RWPE1 cells is due to polyploidy cells driven by chromosomal instability as shown previously [25]. When control and Pim1-expressing cells were grafted in nu/nu nude mice, no tumors formed. H&E stain however showed increased cellularity in Pim1-expressing RWPE1 cells (Figure 3B) and immunohistochemical analysis for Ki67 confirmed elevated proliferation in the tissues of this group (Figure 3C). Apoptosis was also modestly reduced in the Pim1-expressing RWPE1 xenografts as shown by immunostaining for activated Caspase 3 (Figure 3C). These results indicate that although Pim1 could enhance proliferation and suppress apoptosis of RWPE1 cells grown as xenografts, it is insufficient to convert these cells to malignancy.

### Pim1 promotes *in vitro *and *in vivo *tumorigenicity of LNCaP and DU145 cells

**Figure 4:**
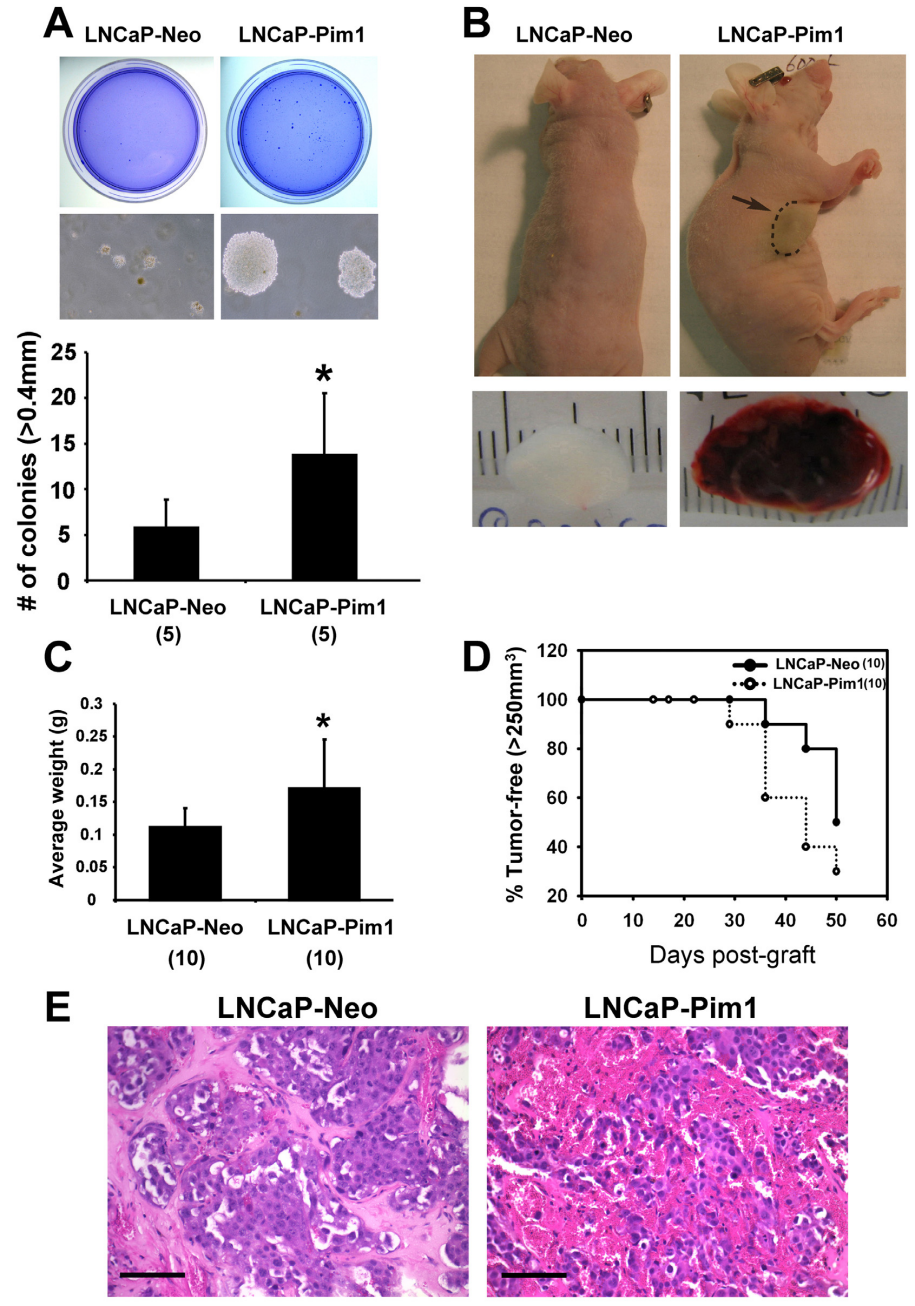
**Pim1 enhances tumorigenicity of androgen-dependent human prostate cancer cells (LNCaP) *in vitro *and *in vivo***. (A) Soft agar assay showed increased *in vitro *tumorigenicity of Pim1-expressing LNCaP cells. When control or Pim1-expressing LNCaP cells were subcutaneously grafted in nude mice, the latter developed larger tumors in size (B) and weight (C). (D) Kaplan-Meier survival analysis demonstrates slightly accelerated tumor onset by Pim1 expression. Numbers in the parentheses indicate the number of replicates or grafts in each group. (E) H&E stains demonstrated that Pim1 expression caused more hemorrhagic phenotype than control. *P < 0.05.

**Figure 5:**
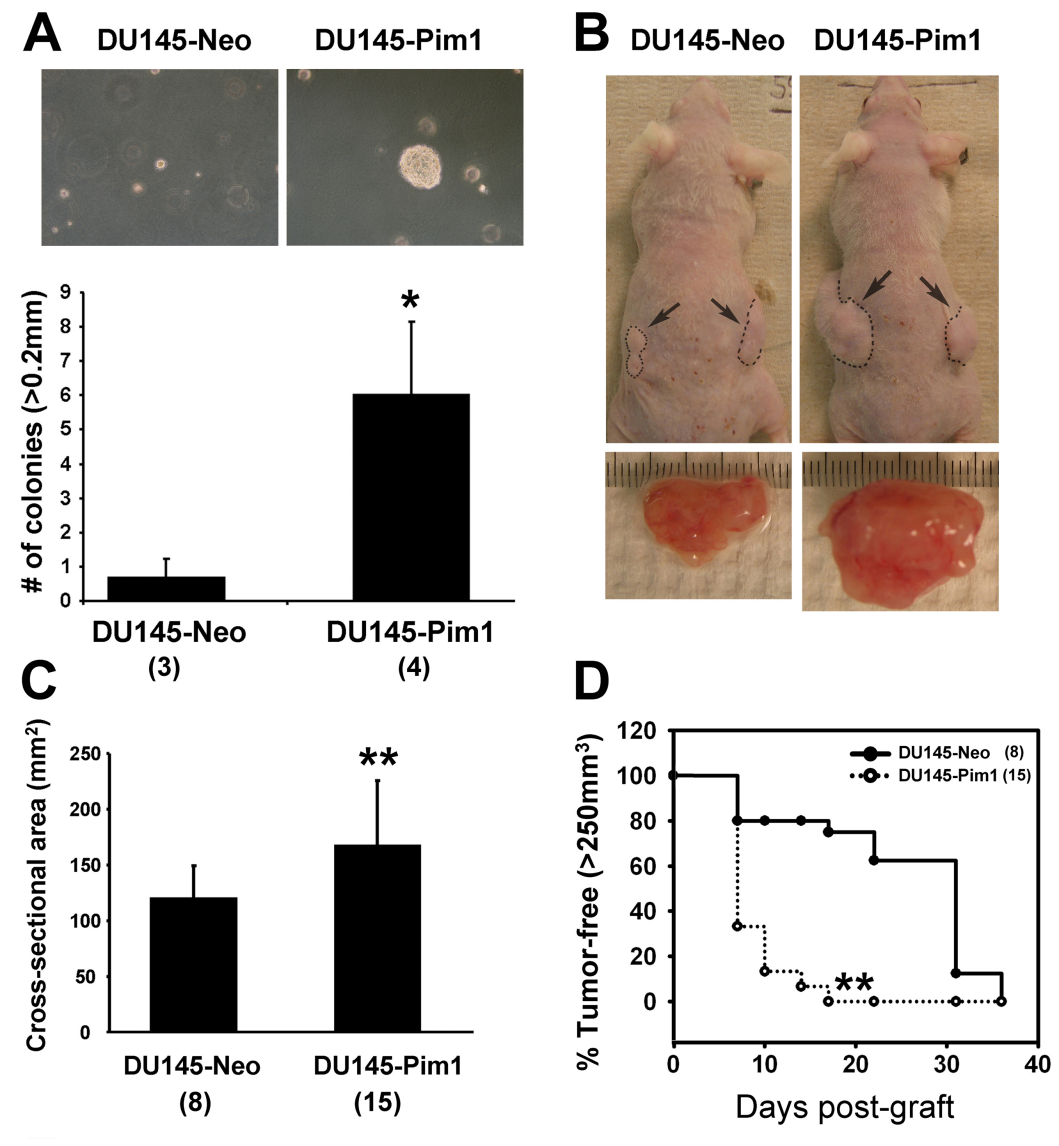
**Pim1 enhances tumorigenicity of aggressive human prostate cancer cells (DU145) *in vitro *and *in vivo***. (A) Soft agar assay shows increased *in vitro *tumorigenicity of Pim1-expressing DU145 cells. When control or Pim1-expressing DU145 cells were subcutaneously grafted in nude mice (nu/nu), the latter developed larger tumor in size (B and C). (D) Kaplan-Meier survival analysis demonstrates significantly accelerated tumor onset by Pim1 expression. Numbers in the parentheses indicate the number of replicates or grafts in each group. *P < 0.01, **P < 0.05.

We next asked whether Pim1 can enhance the tumorigenicity of established malignant prostate cancer cells. We first tested the androgen-sensitive prostate cancer cell line LNCaP. Pim1 expression increased the soft agar colony formation rate of LNCaP cells by ~2 fold and also led to the formation of larger colonies (Figure 4A). When grafted subcutaneously in nude mice, LNCaP-Pim1 cells formed bigger and heavier tumors (Figure 4B and 4C) with shorter latency (Figure 4D). LNCaP-Pim1 tumors were also more noticeably hemorrhagic by both gross and microscopic examination (Figure 4B and 4E). We analyzed proliferation in the tumors by immunohistochemistry for phospho-histone H3. Proliferation was significantly increased in the LNCaP-Pim1 tumors relative to the LNCaP-Neo control tumors (8.8% ± 1.2 versus 3.5% ± 1.3, *P *= 0.003). There was also a trend to reduction in apoptosis as determined by staining for activated caspase 3 (0.35% ± 0.25 in LNCaP-Pim1 tumors versus 0.57% ± 0.58 in LNCaP-Neo tumors). Thus Pim1 can promote the tumorigenicity of LNCaP cells. To extend our findings we also used DU145 cells expressing Pim1 in soft agar assays. Pim1 expression increased DU145 cell colony forming potential up to ~6 fold as well as colony size (Figure 5A). When injected into nude mice, DU145-Pim1 cells also formed larger tumors with a shorter latency (Figure 5B,C and 5D). Thus Pim1 expression can further enhance the tumorigenicity of established tumor cells.

### Androgen receptor signaling does not affect *in vitro *cellular proliferation but its transcriptional activity is induced by Pim1

**Figure 6:**
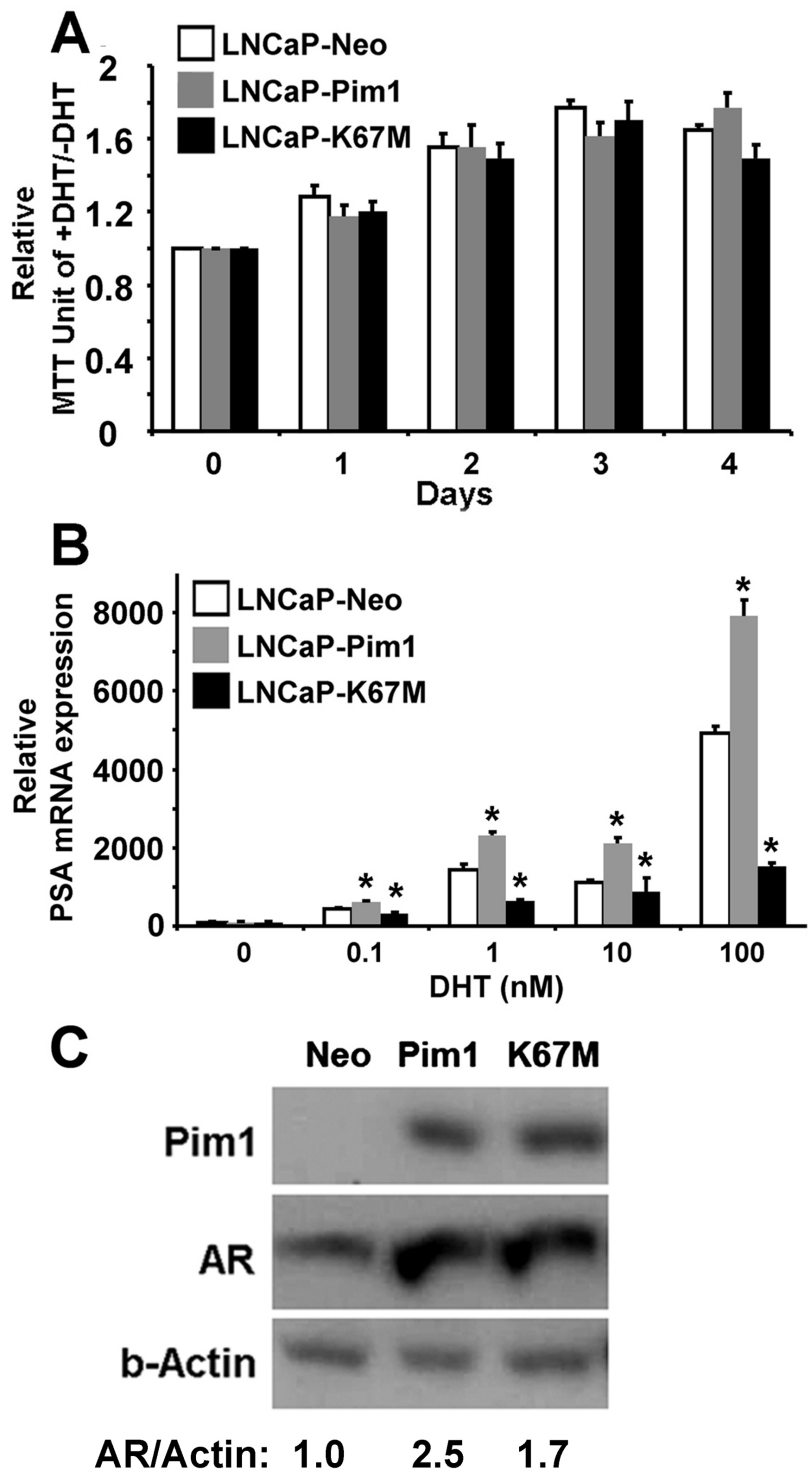
**Role of androgen in proliferation and transcriptional activity of androgen receptor in control and Pim1-expressing LNCaP cells**. (A) Effect of androgen receptor signaling on cell proliferation. Cell proliferation with or without DHT (5α-Dihydrotestosterone) treatment was analyzed in control and Pim1-expressing LNCaP cells. (B) PSA mRNA levels were measured by RT-PCR analysis after the treatment of various dose of DHT. Neo vs. Pim1 or Neo vs. K67M was compared. *P < 0.05. (C) Western blots for Pim1, androgen receptor (AR) and Actin in the indicated cell lines.

Pim1 has been reported to modulate androgen receptor signaling in prostate cancer cells [35,36]. To examine whether androgen receptor signaling affects Pim1 tumorigenic function, we compared androgen-dependent cell proliferation rates between control and Pim1-expressing LNCaP cells. As shown in Figure 6A, expression of Pim1 or a Pim1 kinase-dead mutant (K67M) did not affect androgen-stimulated cell proliferation. AR signaling effects on *in vitro *cell proliferation could be dissociated from other AR signaling functions. Therefore next, we tested androgen receptor transcriptional activity by examining induction of PSA mRNA expression after 5α-Dihydrotestosterone (DHT) treatment. LNCaP-Pim1 cells responded to DHT with significantly higher induction of PSA compared to LNCaP-Neo control cells (Figure 6B). Notably, LNCaP-K67M cells which express the Pim1 kinase-dead mutant showed even lower PSA induction compared to LNCaP-Neo cells (Figure 6B), consistent with the interpretation that the K67M protein functions as a dominant negative mutant. Western blot analysis demonstrated that both Pim1- and K67M-expressing LNCaP cells displayed induction of AR protein expression compared to control LNCaP-Neo cells (2.5 fold and 1.7 fold, respectively) (Figure 6C). Therefore, elevated AR expression can partially explain the increase in PSA levels in LNCaP-Pim1 cells. Other mechanisms are probably operative however, since LNCaP-K67M cells have also increased AR levels but lower PSA expression than control LNCaP-Neo cells (Figure 6B and 6C). These results suggest that Pim1 could promote prostate tumorigenesis by enhancing AR transcriptional activity.

### Pim1 enhances c-MYC transcriptional activity

Pim1 tumorigenic function has been intimately linked to c-Myc in several systems. Pim1 is thought to cooperate with c-Myc to promote tumorigenicity by increasing c-Myc protein expression [21,37] or stimulating the binding of RNA polymerase II leading to increase the transcription of c-Myc target genes [38]. To assess c-MYC transcriptional activity in Pim1-expressing prostate cells we used a c-Myc-responsive 4x-Ebox reporter in luciferase assays. RWPE1-Pim1 cells demonstrated higher c-MYC reporter activity compared to RWPE1-Neo cells (Figure 2A). Furthermore, activity of the c-Myc reporter was suppressed in RWPE1-K67M cells consistent with a dominant negative function of the kinase-dead mutant Pim1 (K67M). This dominant negative action of the K67M mutant is again evident as repression of expression of Cyclin E, a known c-MYC target gene, in RWPE1-K67M cells (Figure 2B).

**Figure 7:**
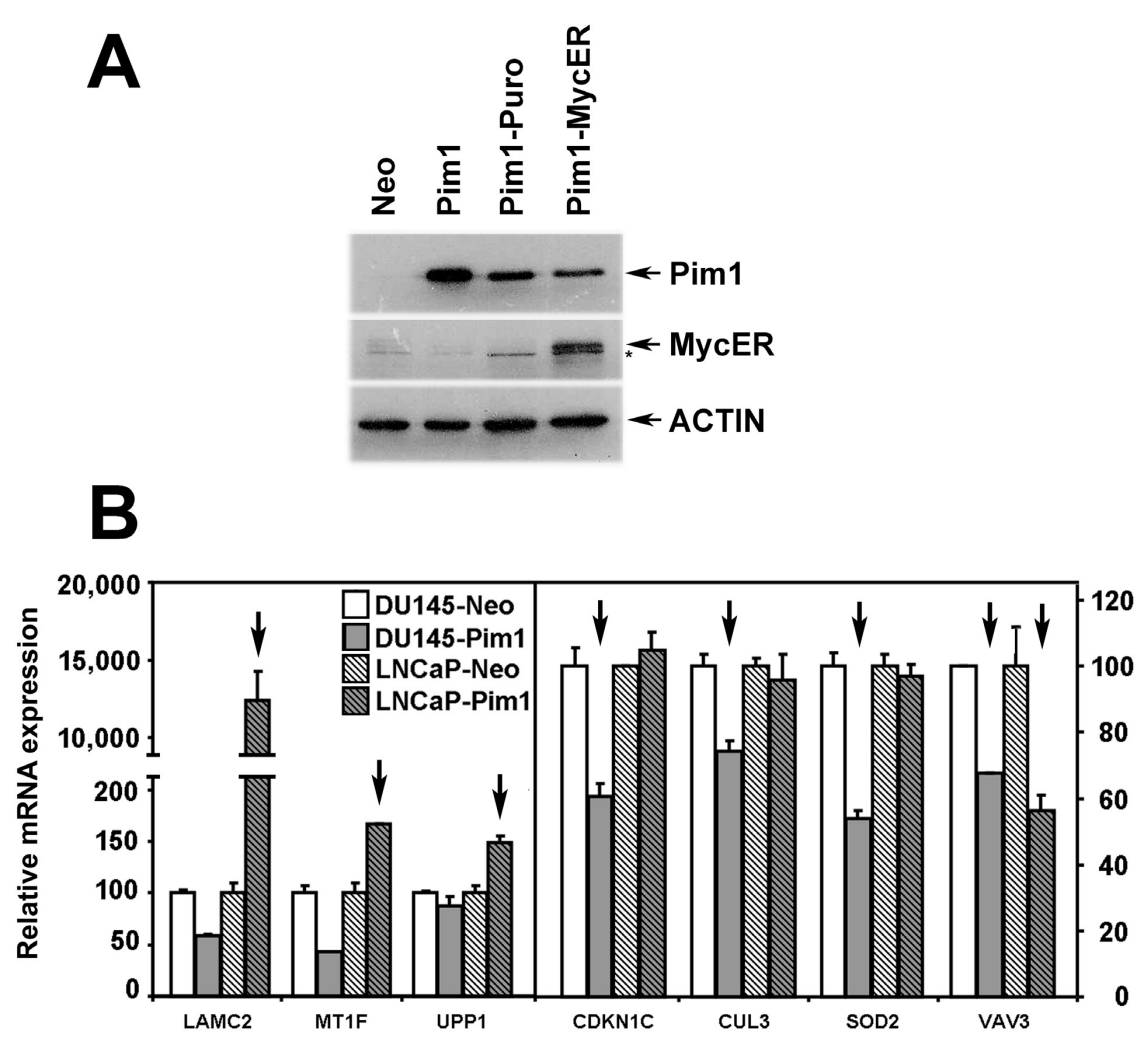
**Examination of gene expression profile using MYC-ER inducible system in RWPE1 cells and its validation of selected genes in LNCaP and DU145 cells**. (A) A MYC-ER inducible system was established in RWPE1 prostate cells and shown is western blot analysis of stable protein expression of MYC-ER, Pim1 and Actin (arrows) in RWPE1 cell lines. * marks a non-specific band. (B) RT-PCR confirmed mRNA expression of several MYC/Pim1 target genes selected from Table 1 in LNCaP-Pim1 and DU145-Pim1 cells. Arrows indicate selected genes that are consistently regulated by Pim1 in DU145 and LNCaP cells.

To obtain a more global view of the possible ability of Pim1 to enhance c-MYC transcriptional activity, we first used an inducible Myc system (MYC-ER, in which c-MYC is fused to a mutant estrogen receptor that responds to 4-hydroxytamoxifen) to identify Myc responsive target genes in RWPE1 prostate cells. We generated stable RWPE1-Neo/MYC-ER and RWPE1-Pim1/MYC-ER cells (Figure 7A). Affymetrix genechip profiling after 24-hr 4-hydroxytamoxifen (4OHT) induction identified 129 c-Myc target genes that were up-regulated or down-regulated in RWPE1-Neo/MYC-ER cells. We then compared these with the genes whose expression is altered by Pim1 expression in the RWPE1-Pim1/MYC-ER (with vehicle treatment) cells. A considerable portion (53 genes, 41%) of the 129 Myc target genes was also altered by Pim1 expression (Table 1). In addition, mRNA expression of some Pim1/MYC target genes in Table 1 was also confirmed in LNCaP and DU145 cells by RT-PCR (Figure 7B) although as expected, there was some variability probably due to the different genetic contexts of these malignant cell lines. *LAMC2*, *MT1F *and *UPP1*, for example, were up-regulated in Pim1-expressing LNCaP cells and genes like *CDKN1C*, *CUL3*, *SOD2 *and *VAV3 *were repressed in Pim1-expressing DU145 and/or LNCaP cells. These results strongly support the notion that Pim1 cooperates with c-Myc in prostate cancer at least in part by modulating c-Myc-transcriptional activity.

**Table 1:** List of common genes altered by Myc induction and by Pim1 expression in RWPE1-MYC-ER cells

Probe Set ID	Gene Symbol	Gene Title	Regulation
209101_at	*CTGF*	connective tissue growth factor	Up
222247_at	*DXS542*	X-linked retinopathy protein-like	Up
201631_s_at	*IER3*	immediate early response 3	Up
202267_at	*LAMC2*	laminin, gamma 2	Up
217165_x_at	*MT1F*	metallothionein 1F	Up
212185_x_at	*MT2A*	metallothionein 2A	Up
213421_x_at	*PRSS3*	protease, serine, 3	Up
209277_at	*TFPI2*	tissue factor pathway inhibitor 2	Up
203234_at	*UPP1*	uridine phosphorylase 1	Up
207275_s_at	*ACSL1*	acyl-CoA synthetase long-chain family member 1	Down
204151_x_at	*AKR1C1*	aldo-keto reductase family 1, member C1	Down
211653_x_at	*AKR1C2*	aldo-keto reductase family 1, member C2	Down
205623_at	*ALDH3A1*	aldehyde dehydrogenase 3 family, memberA1	Down
204942_s_at	*ALDH3B2*	aldehyde dehydrogenase 3 family, member B2	Down
208498_s_at	*AMY1A/-2B*	amylase, alpha 1A/1B/1C/2A/2B	Down
209546_s_at	*APOL1*	apolipoprotein L, 1	Down
39248_at	*AQP3*	aquaporin 3 (Gill blood group)	Down
204820_s_at	*BTN3A2/3*	butyrophilin, subfamily 3, member A2/A3	Down
212067_s_at	*C1R*	complement component 1, r subcomponent	Down
218983_at	*C1RL*	complement component 1, r subcomponent-like	Down
202357_s_at	*C2, CFB*	complement component 2/complement factor B	Down
214164_x_at	*CA12*	carbonic anhydrase XII	Down
209301_at	*CA2*	carbonic anhydrase II	Down
209771_x_at	*CD24*	CD24 molecule	Down
213182_x_at	*CDKN1C*	cyclin-dependent kinase inhibitor 1C (p57, Kip2)	Down
201428_at	*CLDN4*	claudin 4	Down
219529_at	*CLIC3*	chloride intracellular channel 3	Down
204085_s_at	*CLN5*	ceroid-lipofuscinosis, neuronal 5	Down
201117_s_at	*CPE*	carboxypeptidase E	Down
201372_s_at	*CUL3*	cullin 3	Down
218986_s_at	*DDX60*	DEAD (Asp-Glu-Ala-Asp) box polypeptide 60	Down
204646_at	*DPYD*	dihydropyrimidine dehydrogenase	Down
207793_s_at	*EPB41*	erythrocyte membrane protein band 4.1	Down
204569_at	*ICK*	intestinal cell (MAK-like) kinase	Down
206785_s_at	*KLRC1/2*	killer cell lectin-like receptor subfamily C, member1/2	Down
207723_s_at	*KLRC3*	killer cell lectin-like receptor subfamily C, member 3	Down
207761_s_at	*METTL7A*	methyltransferase like 7A	Down
209596_at	*MXRA5*	matrix-remodelling associated 5	Down
205220_at	*NIACR2*	niacin receptor 2	Down
213075_at	*OLFML2A*	olfactomedin-like 2A	Down
203895_at	*PLCB4*	phospholipase C, beta 4	Down
202917_s_at	*S100A8*	S100 calcium binding protein A8	Down
208607_s_at	*SAA1/2*	serum amyloid A1/A2	Down
213988_s_at	*SAT1*	spermidine/spermine N1-acetyltransferase 1	Down
211361_s_at	*SERPINB13*	serpin peptidase inhibitor, clade B, member 13	Down
215223_s_at	*SOD2*	superoxide dismutase 2, mitochondrial	Down
203787_at	*SSBP2*	single-stranded DNA binding protein 2	Down
214970_s_at	*ST6GAL1*	ST6 beta-galactosamide alpha-2,6-sialyltranferase 1	Down
202644_s_at	*TNFAIP3*	tumor necrosis factor, alpha-induced protein 3	Down
202687_s_at	*TNFSF10*	tumor necrosis factor (ligand) superfamily, member 10	Down
213293_s_at	*TRIM22*	tripartite motif-containing 22	Down
208596_s_at	*UGT1A1-10*	UDP glucuronosyltransferase 1 family, A1/A3-A10	Down
218806_s_at	*VAV3*	vav 3 guanine nucleotide exchange factor	Down

### c-MYC inhibition by 10058-F4 treatment or RNA interference abrogates *in vitro *cellular growth and alters target gene expression of Pim1-expressing prostate cancer cells

**Figure 8:**
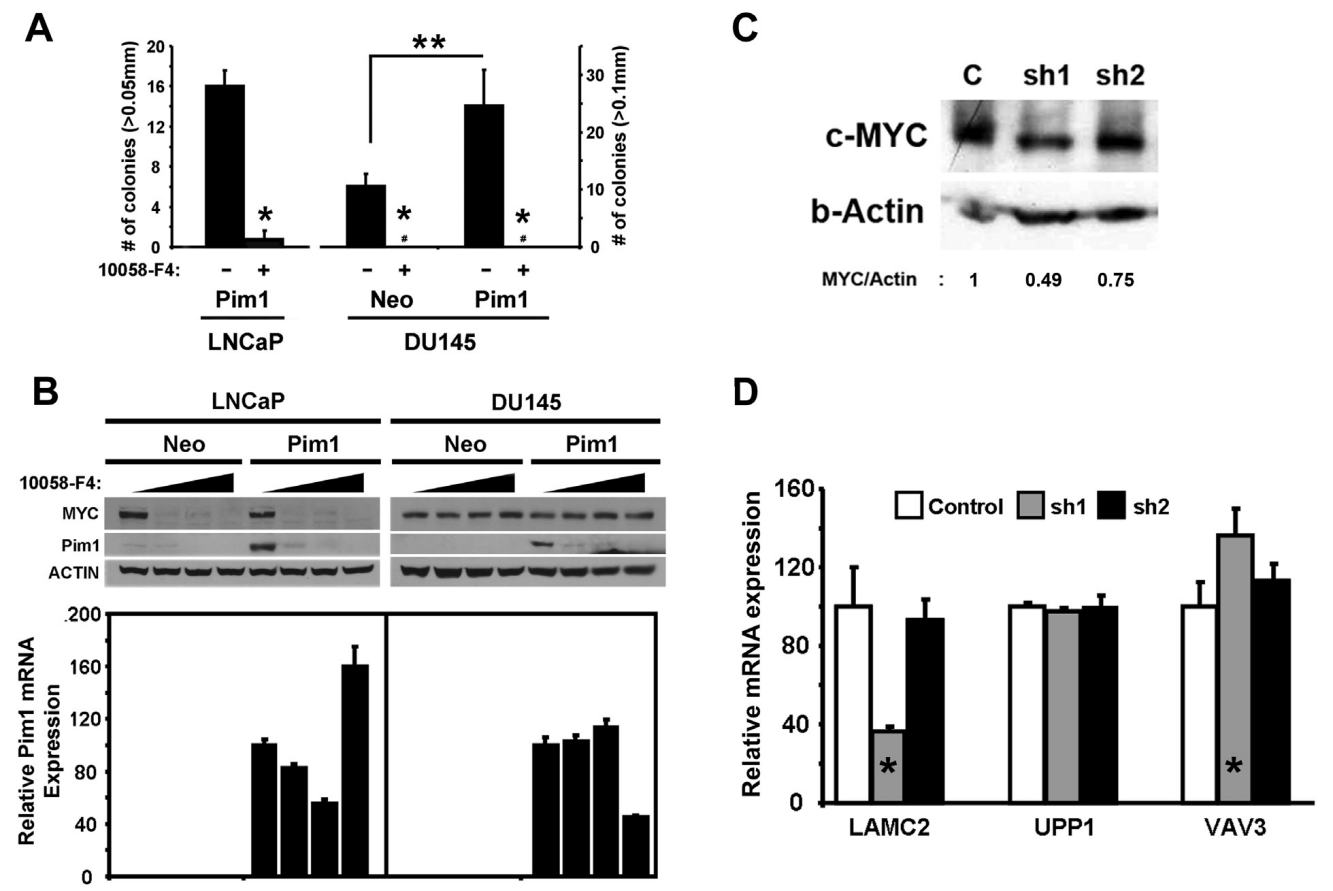
**c-MYC inhibition either by a small molecule inhibitor (10058-F4) or by RNA interference (RNAi) causes *in vitro *growth arrest and alters target gene expression**. (A) c-Myc inhibitor (10058-F4) abrogated colony formation of LNCaP and DU145 cells in soft agar assay. # indicates no colonies. *P < 0.0001, **P < 0.002. (B) Control and Pim1-expressing LNCaP and DU145 cells were treated with different doses (0, 50, 100 and 200 uM) of 10058-F4. Note that 10058-F4 inhibits protein expression of Pim1, but not Pim1 mRNA. (C) Control and two small hairpin RNA constructs (sh1 and sh2) against c-MYC were transfected in LNCaP-Pim1 cells to knock down c-MYC expression. Relative c-MYC expression is shown. (D) Expression of some target genes (*LAMC2 *and *VAV3*) in LNCaP cells was reversed by repression of c-MYC levels in a dose-dependent manner. * indicates altered target gene expression by c-MYC knock-down.

To examine if increased c-MYC activity truly contributes to promote tumorigenicity in Pim1-expressing cells, we inhibited c-MYC activity with 10058-F4, a small molecule c-Myc inhibitor. 10058-F4 is known to inhibit hetero-dimerization between c-Myc and Max, so c-Myc is no longer able to trans-activate its transcriptional target genes [39-41]. In soft agar assays, 10058-F4 dramatically suppressed colony formation of LNCaP-Pim1 and DU145-Pim1 cells (Figure 8A). Next, we treated cells with different doses of the inhibitor (0, 50, 100 and 200 uM). Interestingly, 10058-F4 inhibited c-MYC expression itself in LNCaP cells as shown previously but not in DU145 cells (Figure 8B). This phenomenon, where 10058-F4 treatment reduces MYC protein levels in some cell types but not others, even though it inhibits MYC activity in both types of cells, has been noted previously [40,41].

Remarkably, 10058-F4 also repressed both the endogenous human and transgenic murine Pim1 protein expression in both cell lines (Figure 8B). This effect is not due to a global, non-specific effect on protein expression since neither beta-Actin expression used as a loading control in both cells nor MYC in DU145 cells were changed by inhibitor treatment (Figure 8B). To determine if 10058-F4 treatment affects Pim1 expression at the protein or mRNA levels, we performed RT-PCR to detect the transfected murine Pim1 [24] in LNCaP and DU145 cells. Pim1 mRNA levels were not dramatically changed after inhibitor treatment (Figure 8B), suggesting that 10058-F4 may inhibit Pim1 protein levels via post-translational regulation in addition to c-MYC transcriptional activity.

This novel function of c-MYC inhibitor controlling Pim1 protein expression can not let us examine if decreased tumorigenicity by c-MYC inhibitor is due to inhibited c-MYC activity, repressed Pim1 expression or both. Therefore, to prove that Pim1-induced tumorigenicity is through c-MYC activity, it is necessary to selectively inhibit c-MYC using small hairpin RNA (shRNA). As shown in Figure 8, depending on the levels of c-MYC repression, cells with ~50% c-MYC knock-down (sh1) displayed significant reversal in target gene expression such as *LAMC2 *and *VAV3*, but cells with ~25% c-MYC knock-down (sh2) only showed relatively minor change (Figure 8C and 8D). Overall, these results support the notion that MYC is an important mediator for some of the Pim1 effects observed in this model.

## Discussion

Recent studies have increasingly implicated overexpression of the PIM1 kinase in several human tumors, including lymphomas, leukemia's, gastrointestinal, pancreatic and prostate cancers [9]. In human prostate cancer, whether PIM1 plays a role in tumor initiation and/or tumor progression has not been clearly defined. Some studies have found absent or weak expression of PIM1 in most high-grade prostatic intraepithelial neoplasia (HGPIN) lesions, the putative precursor lesion for prostate cancer [19], consistent with a role for PIM1 in tumor progression, rather than tumor initiation. By contrast, others have noted PIM1 overexpression in a significant fraction of HGPIN lesions [42,43]. There has been limited *in vivo *experimental investigation of Pim1 oncogenic activity in the prostate. One study used, PC3, an aggressive androgen-independent human prostate cancer cell line, to examine *in vivo *tumorigenicity of Pim1. The results demonstrated that tumor growth in Pim1-expressing PC3 cells was accelerated compared to control cells, and this could be partly due to c-MYC protein induction [21]. Our previous study examining tumorigenic potential of Pim1 in the benign human prostate epithelial cell line RWPE1 indicates that Pim1 overexpression alone is not sufficient for the malignant conversion of these cells [25], a finding that was confirmed in the current study. Rather, time-dependent additional genetic events, propelled by chromosomal instability appeared to be required for Pim1-expressing RWPE1 cells to form tumors *in vivo *[25]. These conclusions are supported by our recent observations that Pim1 expression resulted in mild pathological alterations in normal adult mouse prostate epithelial cells using a tissue recombination system, but it greatly accelerated the progression of c-MYC-initiated tumors [44].

Our findings that Pim1 expression could significantly promote tumorigenicity in established human prostate cancer cell lines (LNCaP and DU145) indicate a clear role for Pim1 in tumor progression. The data also showed effects of Pim1 in promoting tumor proliferation and inhibiting apoptosis *in vivo*. Interestingly, these effects of Pim1 are not always obvious in cells grown on plastic tissue culture plates *in vitro*. Notably, both LNCaP and DU145 cells express appreciable levels of Pim1, and it is likely that at least part of the mechanism by which Pim1 expression promotes tumorigenicity in these cells is via enhancing c-MYC transcriptional activity. This hypothesis is supported by the results of the luciferase assays showing that the transcriptional activity of c-MYC was significantly enhanced by Pim1 in a kinase-dependent manner, by gene expression profiling using a MYC-ER inducible system, and by the finding that changes in target gene expression were reversed by c-MYC inhibition when small hairpin RNA (shRNA) against c-MYC was introduced.

Our finding that 10058-F4, a small molecule c-Myc inhibitor could target both Pim1 protein expression and c-Myc transcriptional activity in both androgen-dependent and -independent prostate cancer cells is intriguing and of potential clinical significance. Since Pim1 and c-Myc cooperate to promote the development of lymphoma [16-18] and prostate cancer [19-21,44], targeting both molecules with one drug could dramatically enhance the efficacy of the treatment in cancer patients. However, a limitation of using this particular inhibitor as a drug relates to short half-life because it is rapidly metabolized *in vivo *[45]. Efforts to improve the efficacy of this compound [46] could lead to a potentially important way to target MYC/Pim1-expressing cancers.

## Conclusion

It is important to determine the roles that specifically relevant oncogenes play in the course of tumorigenesis *in vivo *in order to aid appropriate therapeutic targeting. We have shown that Pim1 promotes the tumorigenicity of human prostate cells (LNCaP and DU145) partially by enhancing activities of the MYC and AR signaling pathways. Our findings support the targeting of Pim1 in human cancer and suggest a potential for using the same inhibitor (such as 10058-F4) to target both MYC and PIM1 in human tumors.

## Abbreviations

K67M: mutation at Lysine 67 (to Methionine); 4OHT: 4-hydroxytamoxifen; MycER: 4OHT-inducible Myc-Estrogen Receptor fusion protein; FACS: Fluorescence-activated cell sorting; MTT: 3-(4,5-dimethylthiazol-2-yl)-2,5-diphenyltetrazolium bromide; ATCC: American Type Culture Collection; PBS: Phosphate-buffered saline; DHT: 5α-Dihydrotestosterone; PSA: Prostate specific antigen; SD: Standard deviation; H&E: Hematoxylin and Eosin; Casp3: Activated Caspase 3; shRNA: small hairpin RNA

## Competing interests

The authors declare that they have no competing interests.

## Authors' contributions

JK carried out in vitro and in vivo tumorigenicity experiments, analyzed data and co-wrote the paper. MJR established cell lines and carried out microarray analyses. SAA conceived of the study, participated in its design and coordination and helped to write the manuscript. All authors read and approved the final manuscript.

## Pre-publication history

The pre-publication history for this paper can be accessed here:

http://www.biomedcentral.com/1471-2407/10/248/prepub
